# Supplementary material for: Content-rich biological network constructed by mining PubMed abstracts
Source: BMC Bioinformatics. 2004 Oct 8;5:147. doi: 10.1186/1471-2105-5-147 (PMC528731; doi:10.1186/1471-2105-5-147)
Supplement: Additional File 2 — The original results of the above study (non-essential files are deleted to keep the file size under the limit set by BMC bioinformatics). [file 1471-2105-5-147-S2.bz2 › chilibotAdditionalFile2/dip05/103ID7937747E433/html/MCL1_BCL2L1.html]

 


 **MCL1** and **BCL2L1** 
  
Found 182 abstracts in PubMed, retrieved 05.  
 

 What does Google say? 
 PDF only 
| .edu only 

---

**Interactive relationship** (e.g. stimulation, inhibition, etc)

**Neutral relationship**- gossypol induced apoptosis triggers down regulation of anti apoptosis Bcl 2 members Bcl X  [ **BCL2L1** ]  L, Bag 1 and Mcl 1  [ **MCL1** ] , up regulation of pro apoptosis Bcl 2 member Bak, activation of caspase,, and 9, up regulation of Apaf 1, release of cytochrome c cyto c from mitochondria, and activation of both DFF45 and PARP.  Ref: 12818369 Biochem Pharmacol, 2003

**Non-interactive relationship** (e.g. studied together, co-existance, homology, etc.)

- Remarkably, these events occurred in the absence of any reduction in the expression of the Bcl 2 family members Bcl 2, Mcl 1  [ **MCL1** ]  and Bcl XL  [ **BCL2L1** ] , or any change in the proapoptotic molecules Bad or Bax.  Ref: 12842827 Am J Physiol Gastrointest Liver Physiol< MedlineTA>Am J Physiol Gastrointest Liver Physiol, 2003
- The levels of Bcl X  [ **BCL2L1** ]  L and Mcl 1  [ **MCL1** ]  remain unchanged.  Ref: 12847548 Genes Immun, 2003
